# A program evaluation report of a rapid scale-up of a high-volume medical male circumcision site, KwaZulu-Natal, South Africa, 2010–2013

**DOI:** 10.1186/s12913-015-0904-2

**Published:** 2015-06-18

**Authors:** Adriane Wynn, Claire C. Bristow, Douglas Ross, Inon Schenker, Jeffrey D. Klausner

**Affiliations:** Department of Health Policy and Management, Fielding School of Public Health, University of California, Los Angeles, California; Department of Epidemiology, Fielding School of Public Health, University of California, Los Angeles, California; St Mary’s Hospital, Mariannhill KZN, Johannesburg, South Africa; Operation Abraham Collaborative, Jerusalem, Israel; Division of Infectious Diseases, Center for World Health, Department of Epidemiology, David Geffen School of Medicine and Fielding School of Public Health, University of California, 9911 W Pico Blvd Suite 955, Los Angeles, California

**Keywords:** Male circumcision, HIV, AIDS, South Africa, Uptake

## Abstract

**Background:**

Male circumcision can provide life-long reduction in the risk of acquiring HIV infection. In South Africa, the KwaZulu-Natal Provincial Department of Health committed to rolling out circumcision programs to address the HIV epidemic. The Department of Health enlisted the help of St. Mary’s Hospital in Mariannhill and the Operation Abraham Collaborative.

**Methods:**

St. Mary’s Hospital and the Operation Abraham Collaborative partnered to establish a voluntary medical male circumcision facility, called Asiphile, and to train surgeons, nurses and health clinic staff to serve KwaZulu-Natal.

**Results:**

Over the course of the implementation period, 9,980 circumcisions were conducted at the Asiphile facility. The uptake numbers increased throughout 2010 and 2011 and began to level off as the demand of early adopters may have been met. Uptake spiked during school vacations and staff training sessions. Additionally, 92 % of clients returned for post-operation follow-up and only 2 % of clients experienced any adverse event.

**Conclusion:**

St. Mary’s Hospital and the Operation Abraham Collaborative were able to cooperate and successfully implement a voluntary medical male circumcision facility in KwaZulu-Natal. Although uptake was lower than projected, lessons learned from efforts to overcome challenges in recruitment, transportation, and coordination can help inform and improve new and existing population-based male circumcision programs.

## Background

The efficacy of male circumcision in reducing HIV transmission is supported by a substantial body of evidence, including three randomized controlled trials and numerous observational and ecological studies [1–8]. Circumcision can provide a life-long reduction in the risk of acquiring HIV infection and transmitting human papillomavirus infections, herpes simplex virus type-2, and certain cancers [9, 10]. Despite major advances in the global response to HIV/AIDS, the World Health Organization (WHO) estimates that for every person starting treatment, two people acquire HIV infection [11]. As such, the implementation of evidence-based, combination prevention programs and policies is essential for halting the HIV epidemic. In 2007 the WHO and UNAIDS adopted guidelines that support the scale-up of male circumcision in countries where the prevalence of heterosexually transmitted HIV is high and male circumcision is low [12]. Circumcision interventions should be implemented as part of a comprehensive HIV prevention strategy that includes testing and counseling, treatment for sexually transmitted infections, and the promotion of correct and consistent use of condoms and safe sexual practices [12].

In response to the WHO recommendations and with support from the President’s Emergency Plan for AIDS Relief (PEPFAR), the KwaZulu-Natal Provincial Department of Health committed to rolling out high-volume, voluntary medical male circumcision to address the HIV epidemic. KwaZulu-Natal is a province of South Africa with extremely high rates of HIV incidence [13]. Importantly, the circumcision program moved forward with the support of the Zulu traditional leader, King Goodwill Zwelithini kaBhekuzulu, who recognized the need for evidence-informed, scalable prevention approaches to reduce the risk of HIV and other sexually transmitted infections [14]. The King’s 2009 public directive is reported to have a continued impact on increasing demand for circumcision among the largely Zulu population in KwaZulu-Natal [15].

St. Mary’s Hospital in Mariannhill, which is located outside the city of Durban, KwaZulu-Natal, was asked to contribute to male circumcision expansion efforts [13]. St. Mary’s is the only district hospital in the Western area of the eThekwini Health District and it is the referral hospital for 19 government-supported community primary health care clinics. The patients attending St. Mary’s are predominantly low income, with low access to education and health care services [16]. Additionally, the district served by St. Mary’s has an HIV prevalence rate of 41.1 %, which is the fifth highest among South African districts [16].

St. Mary’s Hospital formed a partnership with the Operation Abraham Collaborative of Jerusalem, Israel, which is a non-profit consortium of nine Israeli and two Senegalese hospitals and health institutions established in response to WHO and UNAIDS request for support in technology transfer of high volume and high quality voluntary medical male circumcision from Israel to African nations. The Operation Abraham Collaborative assisted St. Mary’s staff with the identification and construction of facilities, equipment procurement, and the training of health care professionals to carry out large scale, adult male circumcision. This report describes the implementation and outcomes of the male circumcision program launched at Asiphile (‘Let’s be healthy’ in isiZulu), a community-level clinic created by the Operation Abraham Collaborative-St Mary’s Hospital partnership.

## Methods

Asiphile was created to encourage increased uptake of male circumcision in the catchment area and its mission is two-pronged: to train and mentor clinic staff in male circumcision and to create a high-volume, high quality male circumcision facility to serve KwaZulu-Natal consistent with WHO guidelines and recommendations [17]. Experts from Operation Abraham Consortium and St Mary’s Hospital initiated a clinical training program called SHESHA (‘is Quick, Be Fast’ in isiZulu). The training model was endorsed by the University of KwaZulu-Natal Medical Faculty for continuing medical education for physicians. The international team was composed of surgeons who have conducted thousands of adult circumcisions and who provided on the spot training and mentoring.

Surgeons, nurses and health clinic staff from St. Mary’s Hospital were recruited, trained, and utilized at the Asiphile facility. The new staff were trained to manage daily program operations, perform forceps-guided male circumcisions, educate clients, and carry out monitoring activities using client records, monthly reviews of complication rates, and monthly booking numbers [18]. The staff training and mentoring included seven, two-week sessions with the first occurring on August 2, 2010 followed by six to ten weeks of mentorship after the completion of training. The sessions consisted of both lectures and applied training that incorporated medical simulation models followed by supervised surgeries on patients [18]. The training focused on five main areas including: 1. Surgery, which included local anesthesia, forceps-guided surgery, hemostatis, suturing, and bandaging. Initially, injectable bupivicaine was administered alone, which was the local anesthesia routinely used by the hospital managing the clinic; however, this procedure was updated to include a mixture of lidocaine and bupivicaine in accordance with WHO guidelines. 2. Client and physician safety and quality assurance. 3. Productivity as measured by the number of circumcisions performed per week; 3. Monitoring client two-day and seven-day follow-up; and 4. Patient education. Adverse events were classified using the standardized protocol from PEPFAR [19].

A needs assessment of available staff, equipment, surgical consumables, supply chain, and physical space was conducted and helped determine the location of the Asiphile clinic. The facility was chosen largely because of its proximity to St. Mary’s hospital, which could allow for the convenient referral of clients between Asiphile and St. Mary’s. Additionally, staff could easily rotate between both facilities. Asiphile became a four-bed surgical clinic, converted from a small industrial facility, which was designed for storage or light engineering, located in Pinetown, a suburb of Durban [13]. Asiphile was designed to enable a team of three doctors, 12 nurses, two counselors, and one administrator to provide up to 50 procedures daily reaching a total of 17,000 male circumcisions annually. Asiphile became operational and began seeing clients during the first two-week staff training session in August 2010 [16]. Thereafter, to prevent surgical staff fatigue, medical staff was rotated every 2–3 weeks between St. Mary’s Hospital and Asiphile. The rotating staff included 10 trained clinicians who were physicians and surgeons employed by the hospital. Staff sharing proved beneficial in that it allowed for a large number of trained and flexible staff whose levels could increase or decrease depending on the daily demands of the clinic.

The male circumcision program was targeted at HIV sero-negative men aged 15–49 years in a catchment area near the clinic of 200,000 men. In terms of patient satisfaction, voluntary surveys were not collected; however, complaints or suggestions were accepted according to St. Mary’s hospital protocols. No incentives were provided for receipt of circumcision-related services. To attract clients, community mobilization activities were undertaken at schools, taxi ranks, and industrial production facilities where men worked. Asiphile counselors were sent to taxi ranks on weekends to distribute t-shirts, posters, and cards with Asiphile branding and information about the benefits of male circumcision. Often those activities were conducted in tandem with HIV testing. Temporary testing sites were advertised and men that tested HIV negative were referred to the male circumcision clinic. Men that tested positive for HIV were referred to St. Mary’s Hospital for further assessment. While HIV testing was encouraged, it was a not a requirement for receipt of services at Asiphile.

Additionally, vans were organized to transport groups of 10–15 men from local primary health care clinics to the surgical site location and back to the clinic. Staff met with the governing bodies of local schools to arrange educational sessions with teachers and students. Finally, in 2011 Asiphile received a grant to advertise circumcision services in local newspapers and the radio in both Zulu and English.

Use of data from systemic monitoring of male circumcision scale-up (SYMMACS) received IRB approval from the Human Research Ethics Committee at the University of Witwatersrand. The use of de-identified, programmatic data from the Asiphile project was also approved by the University of California, Los Angeles Institutional Review Board in October 2014. Patients provided written informed consent prior to receipt of services and minors were required to have consent from a parent or guardian to participate.

## Results

**Fig. 1:**
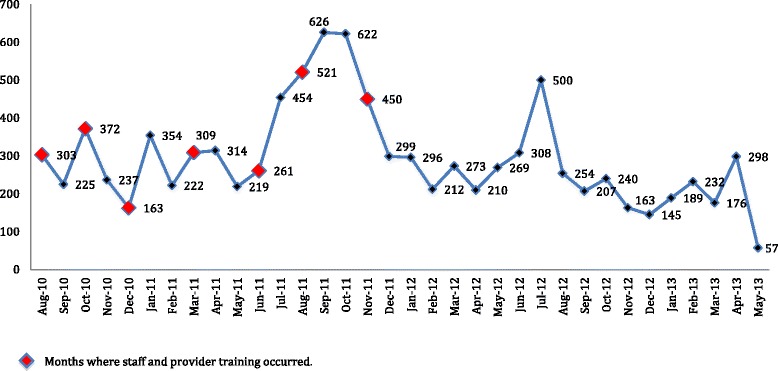
Absolute number of adult circumcisions performed by month at Asiphile, South Africa in 2010–2013

Over the course of the 35-month implementation period, Asiphile conducted 9,980 male circumcisions. The uptake numbers steadily increased throughout 2010 and 2011; however, monthly uptake numbers began to level off and decrease in 2012 and 2013 as demand was met by early adopters. As seen in Fig. 1, the number of circumcisions performed fluctuated ranging from 626 in September 2011 to 57 in the final month of operation. The numbers spike in June through October and show a mild increase in January. Those periods correspond with school vacations that take place beginning in June and again in December and January. Additionally, staff training sessions appear to have had a booster effect. Those trainings took place during August, October and December of 2010; and March, June, August, and November of 2011. Table 1 provides the monthly and yearly totals of male circumcisions performed from July 2010 to May 2013. During the first five months, 1,300 male circumcisions were performed with an average of just under 260 per month. In 2011, the facility conducted 4,651 male circumcisions with a monthly average of 387. Compared with 2011, in 2012 the number of male circumcisions decreased to 3,077, with numbers ranging from 145 in December to 500 in July. The monthly average for 2012 was 256 male circumcisions. In 2013, Asiphile conducted 952 male circumcisions over the course of five months until its closure in May 2013.

**Table 1:** Uptake of adult circumcision by month at Asiphile 2010−2013

	January	February	March	April	May	June	July	August	September	October	November	December	Annual Total
2010							0	303	225	372	237	163	1,300
2011	354	222	309	314	219	261	454	521	626	622	450	299	4,651
2012	296	212	273	210	269	308	500	254	207	240	163	145	3,077
2013	189	232	176	298	57								952

Post-surgical follow-up was conducted in accordance with WHO guidelines [20, 21]. Notably, among all those receiving male circumcision, 92 % of clients returned for their recommended post-operation follow-up on day two and 65 % returned for their follow-up on day seven. Additionally, there were no moderate or severe adverse events and only 2 % of Asiphile clients experienced any adverse event. Adverse advents were evaluated during follow-up and were calculated as the proportion of individuals with any adverse event divided by all individuals that returned for follow-up on day two.

## Discussion

We summarized the results of the implementation and training of an adult male circumcision initiative in KwaZulu-Natal, South Africa. The male circumcision program operated for 35 months and conducted nearly 10,000 procedures. Over the course of its operations, Asiphile generated high procedure outputs with an extremely low rate of minor adverse events.

As expected in any new program, Asiphile faced a number of challenges. The program was unable to meet its goal of 50 procedures per day and the total number of circumcisions performed (9,980) represents only 5 % coverage in the catchment area of 200,000 men; however, these results are similar to other programs in Africa [22]. According to staff, the lower than expected output was largely due to the challenges in identifying and recruiting new clients and the low level of resources allocated to social marketing efforts.

First, while Asiphile was only a ten-minute walk to the transportation hub in Pinetown, that distance posed a barrier for some men. In response to that barrier, the clinic’s outreach workers organized vans to transport batches of 10–15 men from local primary health care clinics to the surgical site location and back to the clinic. Next, while Asiphile was the only permanent site providing male circumcision in the district at the time, a number of other organizations were offering male circumcisions through temporary mobile sites. Those sites were more convenient for patients, but they did not provide a stable location for post-procedure follow-up. Further, competition for new clients generated tension among the organizations offering male circumcision. Beginning in late 2012, meetings were held between the stakeholders to divide up geographic sections of the district. Those divisions ameliorated some of the tension and allowed for organizations to more efficiently target clients. Despite these efforts, engaging a population of young men with limited or no experience in the conventional healthcare system proved difficult. The numbers steadily climbed in 2010 and 2011, possibly due to the early adoption of the procedure by some, but by 2012 the remaining target population was not easily recruited. While clinic staff was consulted about possible reasons for the decline in demand, an important limitation to this report is the lack of information about individual demographic and socioeconomic factors. Further study of the individual characteristics that may serve as barriers or facilitators to circumcision uptake is needed.

Finally, according to staff, Asiphile’s closure in May 2013 was largely due to reductions in external funding. The primary contributor to the Asiphile male circumcision services was PEPFAR, which has been undergoing a country-by-country strategic transition from primary support for the response to HIV/AIDS to country ownership. In 2011, Ambassador Eric Goosby announced that PEPFAR would move from directly funding prevention and care programs to providing technical assistance and capacity building to the South African government [23–25]. Additionally, in August 2012 an announcement was made that PEPFAR funding to South Africa would decrease by half by 2017 [26]. In recent years, the South African government has assumed a greater role in the management and financing of national HIV/AIDS efforts and has invested nearly $1.5 billion [27]. As a result of these changes, some private and non-profit programs originally primarily funded by PEPFAR needed to transition services and patients to South African government-run programs and health care centers [26].

While Asiphile is no longer serving clients, the investments and training made by Operation Abraham Collaborative and St. Mary’s Hospital continue to benefit the KwaZulu-Natal Ministry of Health’s efforts to provide male circumcision to its population. As the first male circumcision provider in the vicinity, Asiphile was able to scale-up an evidence-based, HIV prevention service that was previously unavailable to the high-risk population found in KwaZulu-Natal. Although decreases in HIV incidence have not been measured, several disease modeling studies suggest that in such HIV hyperendemic settings one future HIV infection is prevented for every 5 males circumcised [16, 26]. Based on this simple model, it is possible that Asiphile prevented about 2,000 new HIV infections and generated substantial future savings in HIV treatment and care costs.

## Conclusions

In conclusion, Asiphile made an important and sustained contribution to HIV prevention and male circumcision scale-up in KwaZulu-Natal. Further, the lessons learned from the efforts to overcome difficulties in recruitment, transportation, and coordination can help inform and improve new and existing population-based male circumcision programs. More work, however, is necessary to increase infrastructure and human resources capacity, and to understand individual and organizational characteristics that may be associated with demand for circumcision services [28].

## Abbreviations

PEPFAR: President’s Emergency Plan for AIDS Relief
UNAIDS: The Joint United Nations Programme on HIV/AIDS
WHO: World Health Organization
